# Towards resilience: investigating resources for enhancing climate resilience in health care facilities in low and middle-income countries. a scoping review

**DOI:** 10.1186/s12913-026-14089-x

**Published:** 2026-02-06

**Authors:** Zia Haider, Joacim Rocklöv, Peter Berry, Kiran Jobanputra, Kristie L. Ebi, Tuan Anh Ngo, Patricia Nayna Schwerdtle

**Affiliations:** 1 Faculty of Medicine and University Hospital, Heidelberg Institute of Global Health (HIGH), Heidelberg University, Heidelberg, Germany; 2 The Interdisciplinary Centre for Scientific Computing, Heidelberg University, Heidelberg, Germany; 3 Health Canada, Climate Change and Health Office, Ottawa, ON Canada; 4 The Climate Action Accelerator, Geneva, Switzerland; 5 Centre for Health and the Global Environment, University of Washington, Seattle, USA; 6 Goethe University Frankfurt, Frankfurt, Germany; 7 Faculty of Environment, University of Waterloo, Waterloo, ON Canada

**Keywords:** Climate change, Climate resilience, Low and middle-income countries, Healthcare facilities, Health systems, Resources

## Abstract

**Background:**

Climate change is increasingly impacting healthcare facilities worldwide, with those in low and middle-income countries generally facing high risks due to higher vulnerability and lower adaptive capacity. This scoping review examines resources to strengthen the climate resilience of healthcare facilities in low and middle-income countries.

**Methods:**

We used Arksey & O’Malley (2005) and Levac et al. (2010) scoping review methodology to systematically search four academic databases (Scopus, PubMed, CINAHL, and Lilac), as well as Google Scholar, and the grey literature (websites from the World Health Organization, Green Climate Fund, Global Adaptation Mapping Initiative, Health Care Without Harm, and the Global Environmental Facility) between 2015 and 2024. A coding framework guided the data extraction, which was analyzed quantitatively and qualitatively (Braun and Clarke Thematic analysis).

**Results:**

We identified 33 resources for building the climate resilience of healthcare facilities in low and middle-income countries. The resources varied in their intended audience, composition, and purpose. They produced various outputs (i.e., guidelines, checklists, or adaptation plans) that are presented in a typology to allow users to select the best resource or tool for their purposes. Our analysis identified 14 domains for building climate-resilient healthcare facilities in low and middle-income countries. We present a range of indicators under each domain for measuring the climate resilience of healthcare facilities to enable monitoring and evaluation of climate resilience-building efforts.

**Conclusion:**

There are a growing number of diverse resources that can be used to build climate resilience at the healthcare facilities level in low and middle-income countries. This scoping review offers a comprehensive typology of 33 resources that vary in their focus and application, enabling stakeholders to identify and apply the most appropriate tools for their specific contexts. The 14 key domains identified by this review and associated indicators provide a structured framework for assessing and enhancing climate resilience efforts within healthcare facilities. By offering a range of action-oriented resources and measurable indicators, this review supports ongoing efforts to safeguard healthcare services in low and middle-income countries against the growing impacts of climate change, contributing to the global discourse on sustainable, climate-resilient health systems.

**Supplementary Information:**

The online version contains supplementary material available at 10.1186/s12913-026-14089-x.

## Background

 Climate change is a threat multiplier [1, 2], aggravating underlying vulnerabilities, worsening human health, and disrupting health systems’ abilities to deliver quality and continuous care [3–5]. Health systems comprise the institutions, people, and resources organized to improve population health [6], whereas health care facilities (HCFs) are the operational units, such as hospitals, clinics, and primary care centres, responsible for delivering essential services [7]. Climate resilience in HCFs ensures these facilities can continue providing essential services during extreme weather events [8], protecting vulnerable populations [9, 10], and managing the heightened demand associated with climate-related health issues [4, 11].

Climate risks affect HCFs across short and long-term time scales, from acute hazards such as storms or extreme heat to gradual shifts in temperatures and precipitation patterns [12]. These hazards operate through four main climate drivers: rising air and ocean temperatures, sea level rise, ocean acidification, and more frequent extreme weather events [13, 14]. Such impacts damage health infrastructure [15], disrupt service delivery, and increase the burden of climate-sensitive diseases [12]. HCFs thus serve as the first and last line of defence during climate-related hazards [4, 16], ensuring essential services, infrastructure, and equipment to support them [17].

In Low and middle-income countries (LMICs), HCFs face heightened vulnerability to climate change because of inadequate infrastructure [18], scarce resources [19, 20], and higher exposure to environmental hazards [21]. These weaknesses are magnified during emergencies when demand for care spikes [22, 23]. In LMICs, HCFs are generally unprepared for climate-related hazards [7, 21]. High-income countries (HICs) also face substantial challenges due to climate change, albeit in different forms. Despite shared global challenges, LMICs often experience more severe climate impacts and have fewer adaptive capacities [18, 21]. Strengthening HCF’s climate resilience in such a context is therefore essential for protecting community health, ensuring service continuity, and maintaining affordability [16].

Global commitments, such as the Sendai Framework for Disaster Risk Reduction 2015–2030 [24] and the COP26 Health Programme [25, 26], have emphasized safe, climate-resilient, and low-carbon health systems. Since COP26, evidence has been generated on both impacts and effective adaptation [27]. However, much of the evidence and guidance remains focused on HICs [21, 28, 29]. This imbalance highlights the persistent gap in practical, context-specific knowledge for HCFs in LMICs, where local realities such as inadequate infrastructure, governance structures, and health workforce limitations demand tailored solutions [21]. Additionally, much of the existing research and tools often operate at the system level rather than the facility level [21], focusing on national or sub-national health policies, governance, and overall system functions, which makes them less directly actionable for HCF managers who are focused on adapting specific facilities to climate-related hazards. HCF-level climate resilience remains underexplored despite being central to climate adaptation efforts.

To address these gaps, this scoping review aimed to identify, describe, and synthesize resources, tools, and guidelines for climate change resilient HCFs in LMICs. Specifically, it seeks to:

1. To identify, describe, and collate available resources, guidelines, or tools for developing climate-resilient HCFs or health systems in LMICs.
2. To explore key interventions and indicators for building climate resilience in HCFs in LMICs.
3. To assess and analyse existing gaps in the implementation of climate resilience activities in HCFs in LMICs.

By consolidating evidence and practical tools, this review provides a foundation for decision-makers, facility managers, and practitioners to adapt global frameworks to the specific needs of LMICs. The findings inform planning and implementation across local, regional, and national levels by highlighting the need to translate global guidance into actionable approaches suitable for LMIC health systems. The review thereby supports informed decision-making and guides the application of the climate-resilience and low-carbon interventions identified in this review.

## Method

### Study design

This scoping review of the scientific and grey literature on climate-resilient HCFs in LMICs used a recognized methodology guided by Arksey and O’Malley 2005 [30], with enhancements from Levac et al. 2010 [31]. This scoping review involved comprehensive searches of academic bases, grey literature, and relevant reports to identify resources that support climate resilience in HCFs within LMICs. The PRISMA (Preferred Reporting Items for Systematic Review and Meta-analysis) [32], guideline informed this scoping review. The study protocol was developed, registered, and made public (OSP: http://osf.io/4yqpg*).*

### Research question

What resources, tools, and guidelines are available to support the development of Climate Change-resilient HCFs in LMICs, and what are the key interventions, indicators, and existing gaps in implementing climate resilience activities in these settings?

### Search strategy

#### Eligibility criteria

The research question was refined using a modified PICO framework (Population, Intervention, Comparison, and Outcome), adopted here as Population/Context, Intervention/Phenomenon, and Outcome (PIO) [33]. This PIO framework provided a structured approach for conceptualizing the review to address the research question (see Table 1) The inclusion and exclusion criteria were then established to identify the most relevant articles to answer the research question (see Table 2).

**Table 1:** Concept conceptualization

Concept	Description
Population/Context	Developing countries, Low-and Middle-Income Countries, Global South
Intervention/Phenomenon	Resources for building climate-resilient and environmentally sustainable health care facilities/ health care systems.
Outcome	Increased resilience of HCFs

**Table 2:** Inclusion and exclusion criteria

Criteria	Inclusion Criteria	Exclusion Criteria
Content	a) Addressing the research question. b) Encompasses all the three key concepts. i. Climate Resilience. ii. Healthcare facilities (HCFs). iii. Resources, tools, or guidelines.	Does not answer the research question. Includes some but not all of the key concepts.
Article Type	Academic: empirical, peer-reviewed, grey literature from reputable resources (PAHOiris, World Health Organization databases (WHOlis and WHOiris), Green Climate Fund (GCF), Global Adaptation Mapping Initiative (GAMI), Healthcare without Harm (HCWH), and the Global Environmental Facility (GEF). Primary research.	Blogs, Editorials, Commentary. Media, Secondary research.
Geographical Focus	Developing countries, LMICs (World Bank criteria).	High-income countries (HICs) (World Bank Criteria).
Time frame	Published between 12.12.2015 (marks the date of the Paris Agreement) and 30.6.2024	Published outside the stated time frame.
Language	Search: English (Spanish & German) (translate relevant articles found in other languages)	The searches were performed without restriction to language. However, searches were performed in English.

### Information sources

To identify the relevant original studies and the grey literature, we systematically searched scientific databases: Scopus, PubMed, CINAHL, Lilacs, and the web search engine Google Scholar. For grey literature, the following seven databases and key websites were selected: PAHOiris for Latin American Studies; WHO library databases (WHOlis and WHOiris); The Green Climate Fund (GFC); Global Adaptation Mapping Initiatives websites (GAMI); The Global Environment Facility (GEF); and the Healthcare Without Harm (HCWH) website. Articles published between 12.12.2015 to 30.06.2024 were eligible. The start date of 2015 was chosen for this scoping review as it marks the year of the Paris Agreement when member states formally committed to addressing climate change on a global scale, including its impact on health systems. This pivotal moment signalled an intensified focus on climate adaptation and resilience, promoting increased development of resources, tools, and guidelines specific to building climate-resilient health systems.

#### Search terms

The search terms were identified, including both medical subject headings and keywords; to identify research studies relevant and addressing the questions (Table 3). An example of a search string is provided below (Box 1).

**Table 3:** Search terms

Concept 1 Climate change adaptation	Concept 2 Health systems	Concept 3 LMICs
Climate change adaptationAdaptation responseDisaster risk reductionSustainable developmentEconomic developmentClimate resilienceEnvironmental sustainabilitySustainable use	Health systemsHealth care facilitiesHealth facilitiesPrimary care clinicsHospitals	Developing countries Global SouthLow-income countries (LICs)Middle-income countries (MICs)Low-and Middle-income countries (LMICs) Least developed countries (LDCs)

**Table:** 

Box 1: Example Search String
“Climate change adaptation” OR “climate change” OR “adaptive responses” OR “disaster risk reduction” OR “sustainable development” OR “economic development” OR “climate resilience” OR “environmental sustainability” OR “Sustainable use” (MeSH terms and “major topic” function were used where supported by the database) AND “Health systems” OR
OR “health facilities” OR “hospitals” OR “primary care clinics” (MeSH terms and “major topic” function were used where supported by the database) AND “Developing Countries” OR “global south” OR “low-income countries” OR “middle-income countries” OR “low-and middle-income countries” OR “least developed countries” (MeSH terms and “major topic” function were used where supported by the database)

### Selection of sources of the evidence

Five researchers conducted exhaustive searches of each information source. Documents were screened against the inclusion and exclusion criteria in two phases; title and abstract and then full text review. An additional researcher was consulted in the case of disagreement.

### Data collection process

Extraction codes were formulated a priori and designed to answer the research question “What resources, tools, and guidelines are available to support the development of climate change-resilient HCFs in LMICs, and what are the key interventions, indicators, and existing gaps in implementing climate resilience activities in these settings?” Data were extracted systematically using an Excel spreadsheet. The data extraction fields included descriptive codes (Author and title, full citation), study design features (study location, research aim, study design, methods), and results related to our main research questions. The data was then interpreted, and where necessary, data extraction was double-checked and clarified.

### Data analysis

The key characteristics of the studies were treated as categorical data and reported as frequencies and percentages. The extracted results were analyzed qualitatively using Braun and Clarke thematic analysis [34], guided by the ten domains of the WHO Operational Framework [11]. Each resource was mapped to one or more of these predefined domains, and the frequency of resources addressing each domain was calculated. Additional subthemes emerging from the data were identified inductively, ensuring a combination of deductive and inductive analysis.

## Results

### Screening

**Fig. 1:**
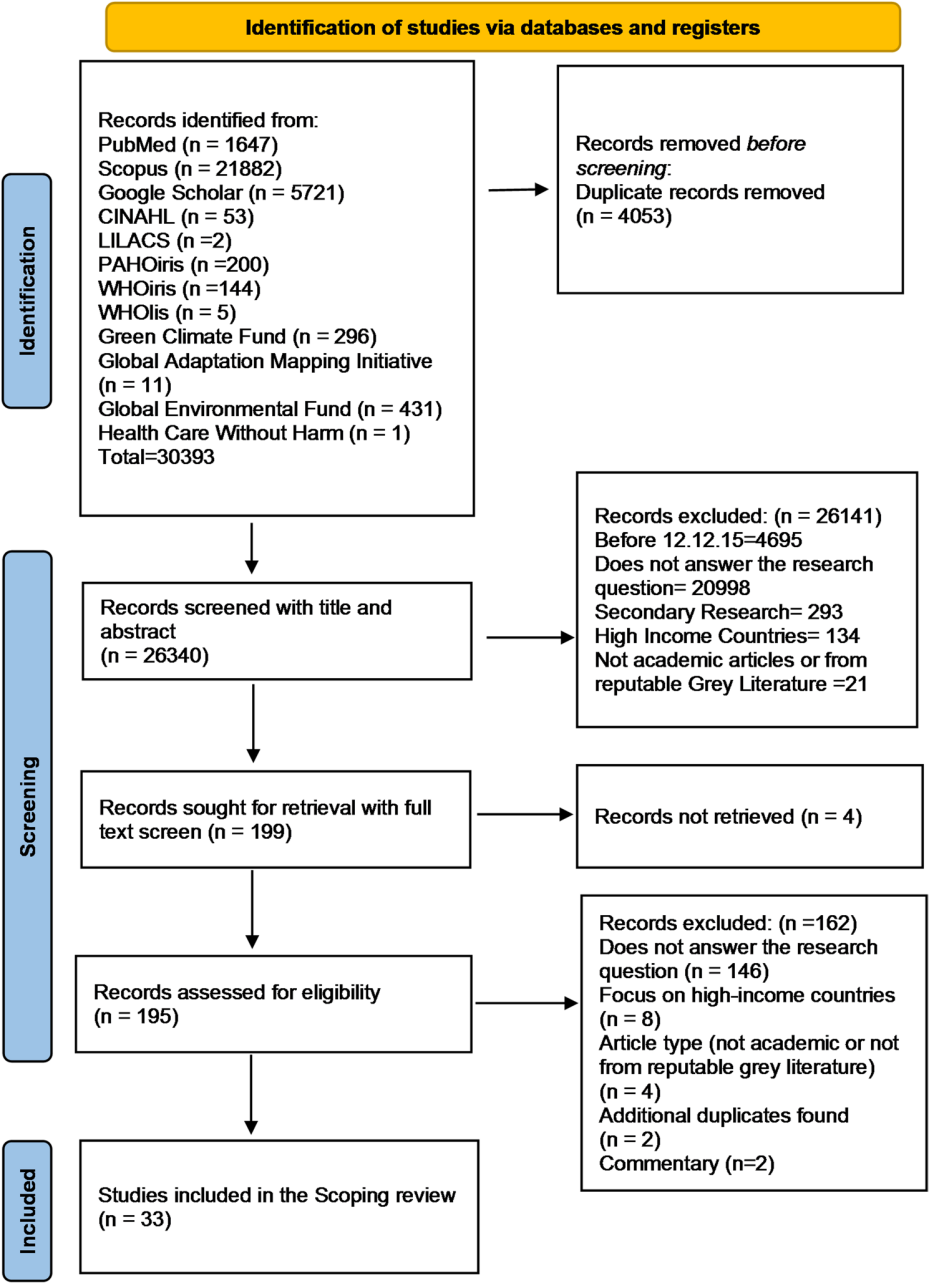
PRISMA screening diagram

In total, 30,393 articles were identified, and 4,053 duplicates were removed using Rayyan systematic review software. After duplicate removal, 26,340 articles were screened by title and abstract. After the first screening stage, 26,141 articles were excluded. In the second screening stage, 195 articles were screened in full text, of which 162 were excluded, and 33 were included in the analysis (Fig. 1).

**Fig. 2:**
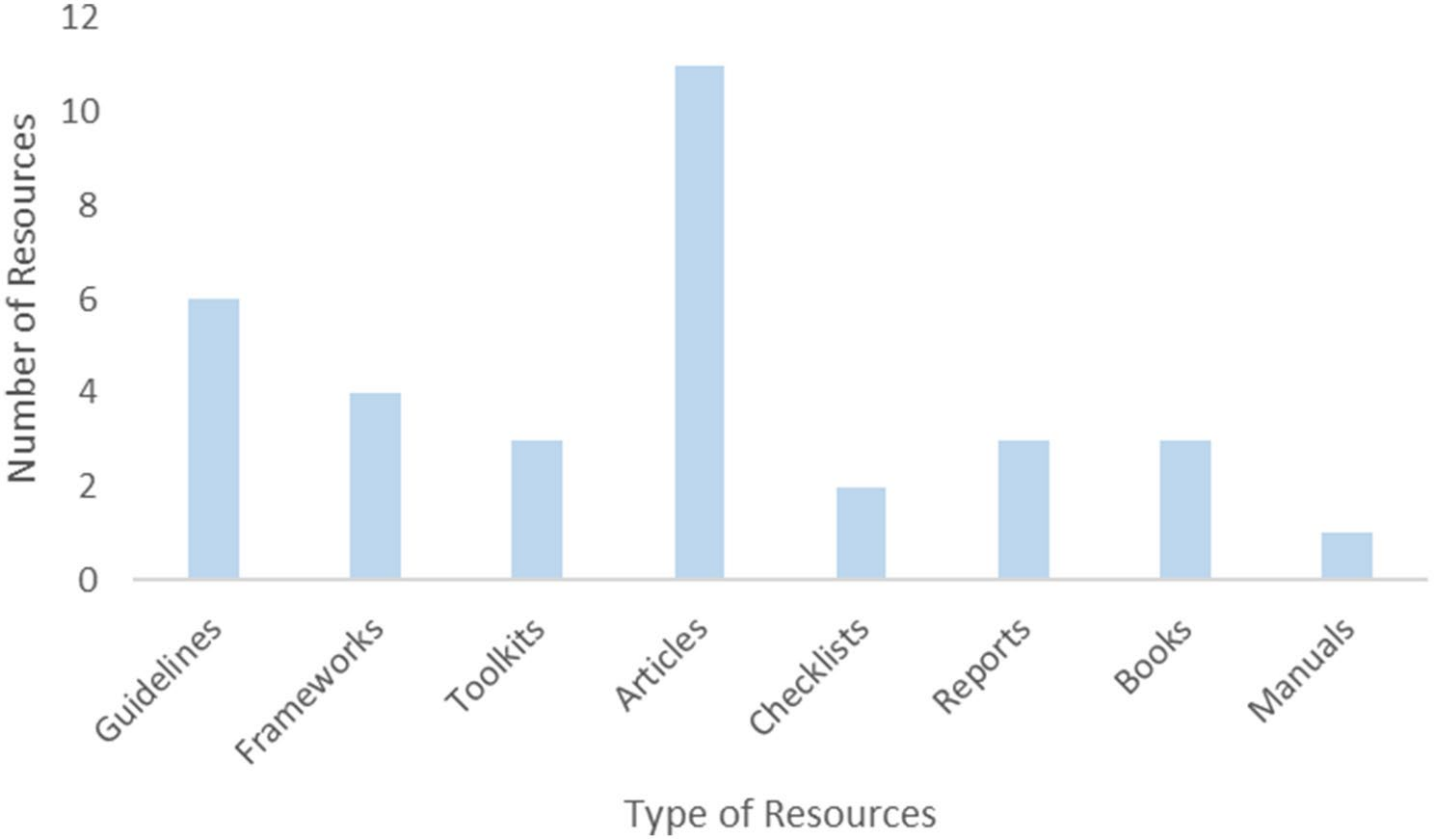
Types of resources for building climate resilience in health care facilities in LMICs

Table 4 gives a list of 33 included resources in the study (for the typology of included resources, see supplementary table). Included resources came in several forms, including academic articles, guidelines, frameworks, toolkits, reports, manuals, and books (see Fig. 2). Of the 33 included resources, around one-third (33%) were academic articles, defined as peer-reviewed journal publications that report original research or reviews. Guidelines for building climate resilience in HCFs were the second most common type of resource, accounting for 18%. Frameworks accounted for 12%; toolkits, reports, and books were equally represented, making up 9% each of the total. Checklists are less frequent, accounting for 6% of resources. Manuals are the least common at 3% of resources, suggesting a potential gap in the availability of detailed operational instructions.

**Table 4:** List of the included resources

Resource	Resource Title	Citation (author, date)	Resource Type
1	WHO Guidance for Climate-Resilient and Environmentally Sustainable Healthcare Facilities	World Health Organization 2020 [4]	Guideline
2	Smart Hospitals Toolkit	Pan American Health Organization 2017 [43]	Toolkit
3	Guidelines for Climate-Resilient and Environmentally Sustainable HealthCare Facilities in Fiji	Ministry of Health and Medical Services 2020 [38]	Guideline
4	Understanding Weather and Hospital Admissions Patterns to Inform Climate Change Adaptation Strategies in the Health Sector in Uganda	Bishop-Williams et al. 2018 [48]	Article
5	Enhancing the sustainability and climate resiliency of health care facilities: a comparison of initiatives and toolkits	Balbus et al. 2016 [59]	Article
6	Caribbean action plan on health and climate change	PAHO 2019 [39]	Report
7	Advanced Operationalization Framework for Climate-Resilient Urban Public Health Care Services: Composite Indicators-based Scenario Assessment of Khon Kaen City, Thailand	Puntub and Grieving 2022 [46]	Article
8	Defining adaptive capacity in healthcare: A new framework for researching resilient performance	Anderson et al. 2020 [49]	Framework
9	Resilience testing of Health Systems: How Can It Be Done?	Rogers et al. 2021 [40]	Article
10	Developing a practical toolkit for evaluating hospital preparedness for surge capacity in disasters	Shabanikiya et al. 2019 [55]	Article
11	Operational framework for building climate resilient health systems	World Health Organisation 2015 [35]	Framework
12	A checklist to improve health system resilience to infectious disease outbreaks and natural hazards	Meyer et al. 2020 [44]	Checklist
13	360° Resilience: A Guide to Prepare the Caribbean for a New Generation of Shocks	Julie Rozenberg et al. 2022 [52]	Report
14	Understanding the resilience of health systems	Blanchet et al. 2020 [50]	Book (chapter)
15	Are we ready for it? Health systems preparedness and capacity towards climate change-induced health risks: perspectives of health professionals in Ghana	Hussey et al. 2019 [56]	Article
16	Climate Change and Health Vulnerability and Adaptation Assessment	Health Canada and World Health Organization 2021[37]	Guideline
17	Health systems resilience toolkit	World Health Organization 2022 [62]	Toolkit
18	Quality Criteria for Health National Adaptation Plans	World Health Organization 2021 [60]	Guideline
19	Impact of extreme weather conditions on healthcare provision in urban Ghana	Codjoe et al. 2020 [53]	Article
20	Towards Climate Resilient and Environmentally Sustainable Health Care Facilities	Corvalan et al. 2020 [16]	Article
21	Climate Sensitive Adaptation in Health Imperatives for India in a Developing Economy Context	Dasgupta 2016 [61]	Book
22	Stress Testing the Capacity of Health Systems to Manage Climate Change- Related Shocks and Stresses	Ebi et al. 2018 [51]	Article
23	Checklists to assess vulnerabilities in healthcare facilities in the context of climate change	World Health Organization 2021 [45]	Checklist
24	Measuring the climate resilience of health systems	World Health Organization 2022 [36]	Report
25	Operational framework for building climate resilient and low carbon health systems	World Health Organization 2023 [11]	Framework
26	Green and Safe Health Facilities Manual	Department of Health Manila 2021 [57]	Manual
27	STAR-H Strategic Toolkit for Assessing Risks in Health Facilities	Pan American Health Organization 2023 [24]	Toolkit
28	Exploring hospitals’ functional preparedness effective factors in response to disasters: a qualitative study in a lower middle-income country	Samei et al. 2024 [54]	Article
29	Iran’s climate resilient health system: Challenges and solutions	Mosadeghrad et al. 2024 [47]	Article
30	Strengthening Health Systems: A Practical Handbook for Resilience Testing	OECD 2024 [41]	Book
31	WASH FIT: A practical guide for improving quality of care through water, sanitation, and hygiene in healthcare facilities	WHO and UNICEF 2022 [42]	Guideline
32	Global Framework for Action 2024–2030 Universal water, sanitation, hygiene, waste, and electricity services in all healthcare facilities to achieve quality healthcare services	World Health Organization 2024 [58]	Framework
33	Operational Guidelines Green, Safe, and Climate-Resilient Health Facilities	Department of Health-Health Facility Development Bureau Philippines 2023 [15]	Guideline

**Fig. 3:**
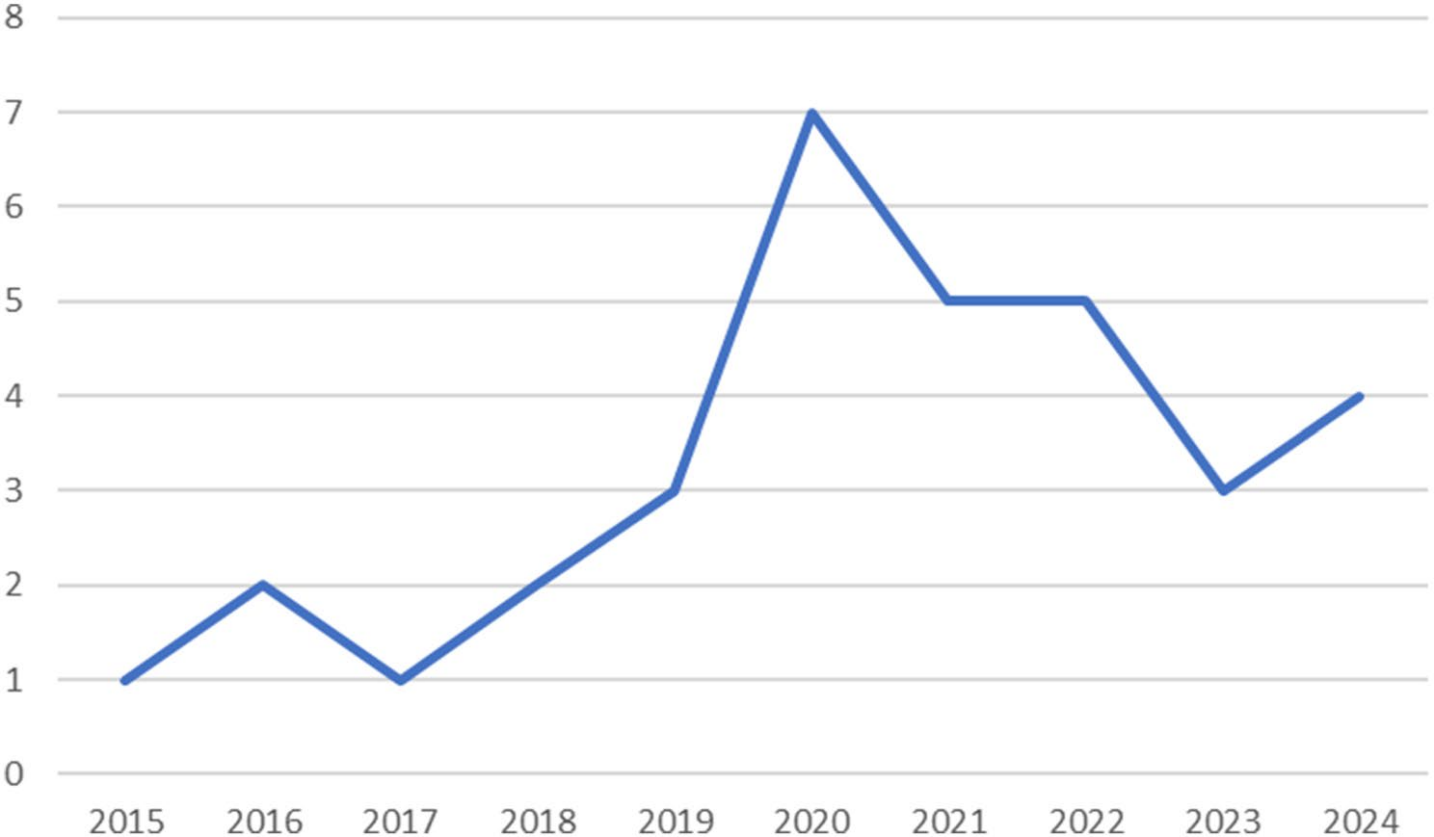
Distribution of included resources by year of publication

Between 2015 and 2019, publication numbers ranged from 1 to 3 per year (see Fig. 3). In 2020, there was a notable increase in publications, with seven resources published that year, accounting for 21% of total resources included in the study. This increase in publications in 2020 likely reflects growing global recognition of climate change as a health threat, reinforced by international momentum in the lead-up to the COP26 Health Programme. This initiative, launched ahead of the 2021 UN Climate Change Conference, resulted in a large number of countries committing to developing climate-resilient and low-carbon health systems, which in turn generated substantial policy attention and research activity. The subsequent years showed stabilization with 3–5 publications annually.

### Geographical map

**Fig. 4:**
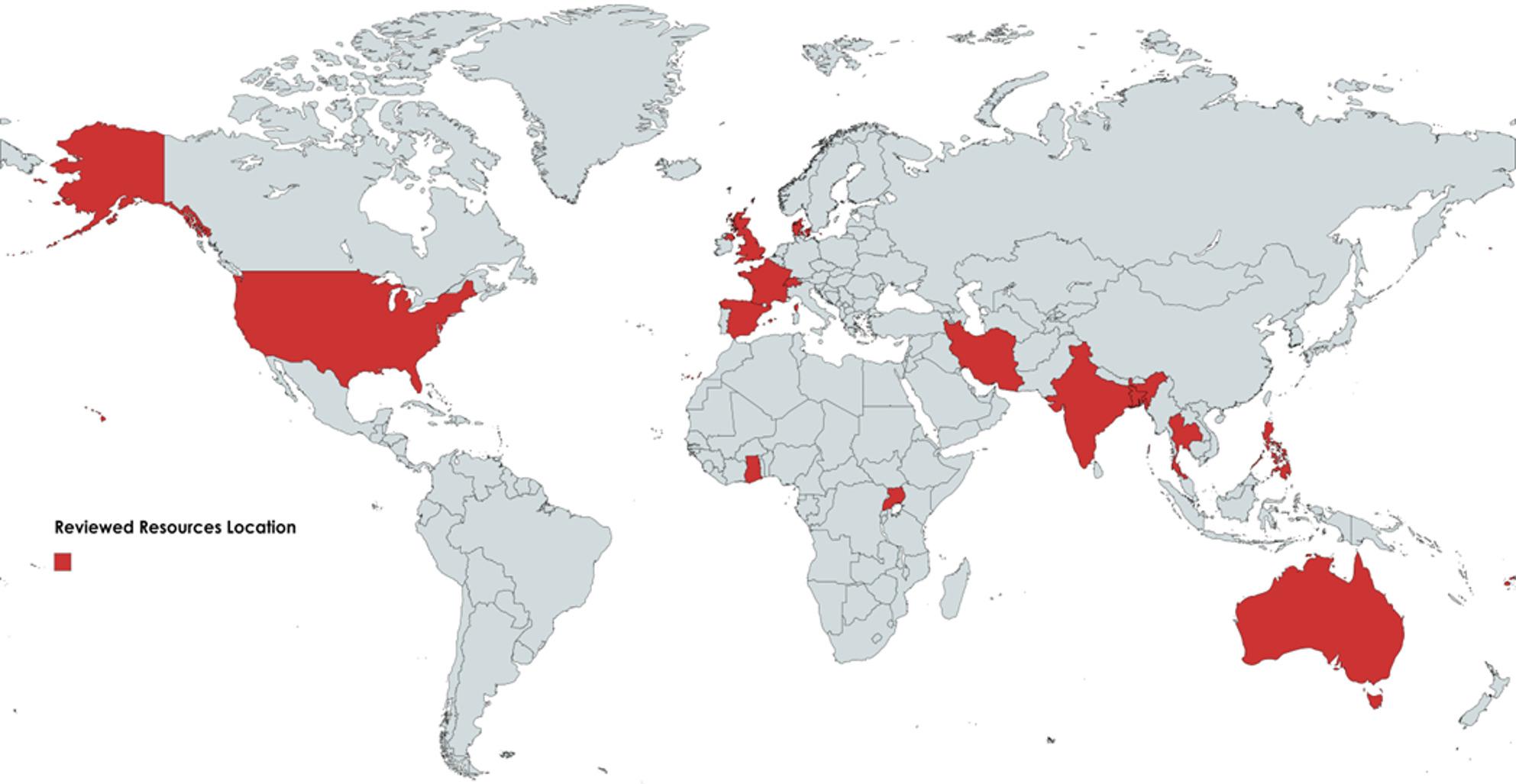
Geographical distribution of included resources by country of authorship/origin (not by area of application)

Figure 4 shows the geographical distribution of the 33 included resources based on the countries of authorship or institutional affiliation, indicating where each resource was developed rather than where it is applied. The resources originated from Ghana, Uganda, Iran, India, Bangladesh, Thailand, Philippines, Fiji, Switzerland (WHO), USA (PAHO and others), Denmark, France, Spain, Australia and UK. A total of 55% of resources (n: 18) were designed for global use or had no defined geographical application. While the remaining 45% of resources (n: 15) are focused on building climate-resilient HCFs in one or more LMICs. Out of these 15 resources, 12 originated from eight LMICs (Ghana, Uganda, Iran, India, Bangladesh, Thailand, Philippines, and Fiji).

### Interventions for building climate resilience in HCFs in LMICs

**Fig. 5:**
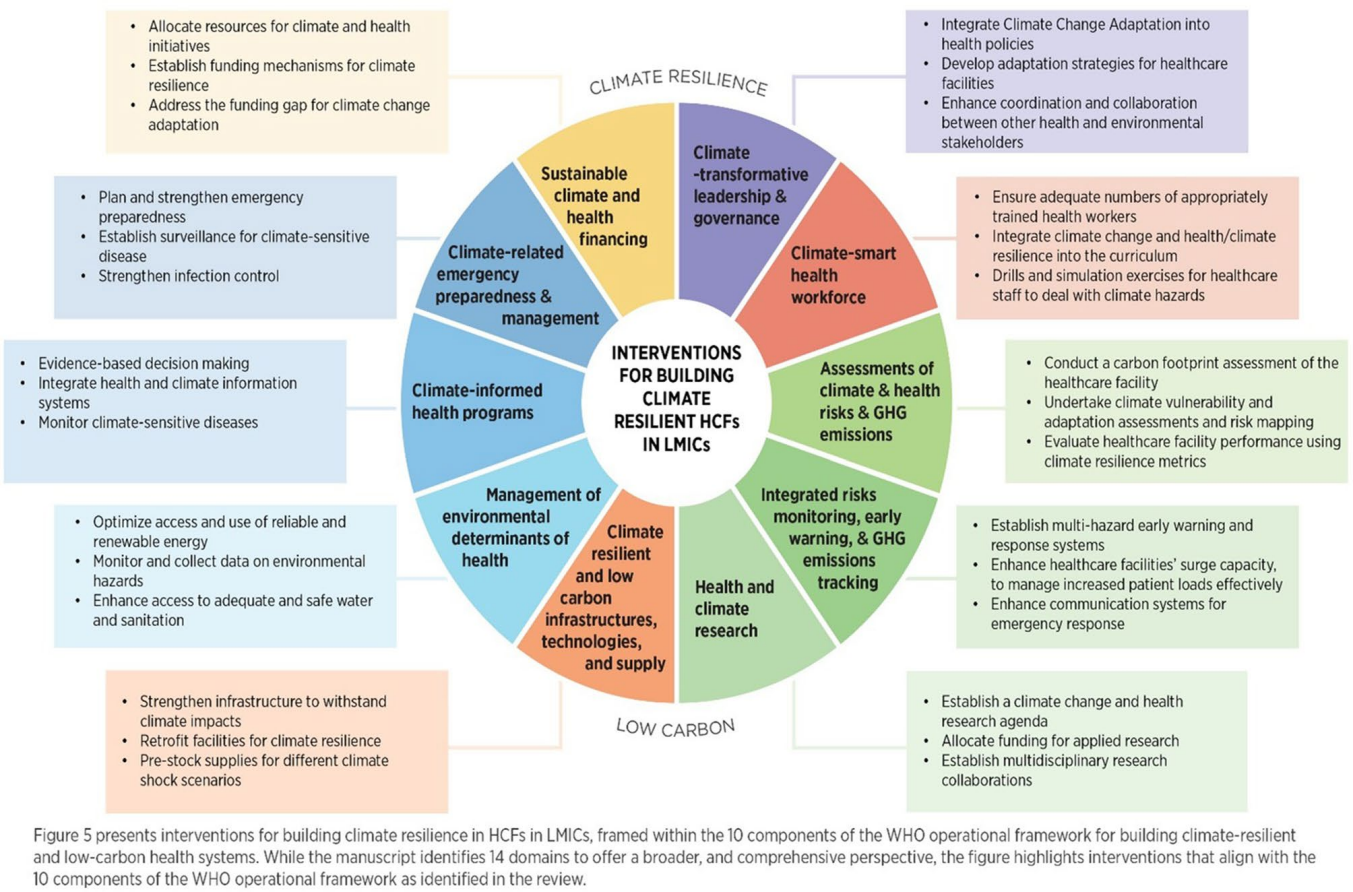
Examples of interventions for building climate resilient HCFs in LMICs

The included resources identified different domains for building climate-resilient HCFs in LMICs, aligning with WHO’s Operational Framework for Building Climate Resilient and Low Carbon Health Systems and associated health system building blocks [11, 35]. The domain refers to a specific area or category of focus within the broader efforts to build climate-resilient HCFs. Each domain represents a distinct aspect or component needed to achieve resilience. While several domains identified in this review align closely with the four components outlined in the “WHO guidance for climate-resilient and environmentally sustainable healthcare facilities”, our analysis extended beyond this framework to incorporate broader governance, financing, and research-related dimensions reflected in the recent WHO operational framework (2023). This approach allowed for a more comprehensive typology relevant to the LMIC context. The identified domains can be categorized into three groups based on their frequency of occurrence across the 33 resources. High-frequency domains include emergency preparedness, response, and adaptation to climate change hazards; access and continuity of health services; climate resilient infrastructure and design of HCFs; policy, planning, and leadership; and health workforce capacity development, education, and training; all of which appeared in more than 70% of the included studies. Moderate-frequency domains found in 50–70% of the included resources include climate change information and data management; water, sanitation, and hygiene (WASH); inter-sectoral collaboration and partnerships; financing and resource allocation; community engagement and participation, energy efficiency, and renewable energy; healthcare waste management and recycling. Last, low-frequency domains, such as climate research and reducing greenhouse gas (GHG) emissions, are included in less than 50% of the resources. Figure 5 (adapted from WHO Operational Framework 2023) provides examples of interventions across the 14 domains discussed above.

### Indicators for building climate-resilient HCFs inLMICs

Indicators are needed to measure progress towards climate resilience. Indicators help provide insights into how well the systems are functioning when faced with challenges from current or future climate change risks [36]. To measure climate resilience, health authorities need information and indicators that are tailored to their needs [37]. This scoping review identified indicators under 14 domains. Table 5 provides example indicators from the reviewed resources. However, these do not represent all the indicators provided by the resources, nor do they cover all the indicators that are available elsewhere. Nine [11, 35–42] out of the 33 included resources provided indicators for climate resilience in HCFs, and four resources [4, 43–45] provided checklists of interventions for building climate resilience.

**Table 5:** Indicators for building climate resilience in HCFs in LMICs

Domain	Number of Resources providing indicators	Example Indicators
Policy, Planning, and Leadership	06 resources [11, 35–37, 39, 40]	1. Development of national strategy on health and climate change resilience of HCFs 2. Established a task force on health and climate change 3. Existence of national adaptation planning for climate resilience
Emergency Preparedness, Response and Adaptation	06 resources [11, 35–37, 39, 41]	1. Developed and implemented contingency plans for the health sector for extreme weather events 2. Risk assessment of current and projected future exposures to climate-related hazards and disasters 3. An early warning system is developed and actively informs HCF to respond quickly to extreme weather events and predict health risks
Climate Change Information and Data Management	05 resources [11, 35, 36, 39, 40]	1. Strengthened health information systems with climate information for early health interventions 2. Health programs integrating information on current and projected climate change risks 3. Information and evidence gathered are used to inform action on priority climate-sensitive health programs
Health Workforce, Capacity Development, Education and Training	08 resources [11, 35, 36, 38–42]	1.HCFs have identified and met minimum staffing needs for operational sufficiency during climate-related disasters and events 2. Health workforce trained to assess potential health impacts due to climate-related hazards 3. Improved staff capacity to provide infection prevention and control measures during climate-related hazards
Climate Resilient Infrastructure and Design of HCFs	06 resources [11, 35, 36, 38–40]	1. The geographical area and population served by HCFs are mapped for extreme weather events and emergencies 2. HCFs are located or retrofitted based on an assessment to avoid high-risk areas and cope better with climate-related risks and hazards 3. HCF assessed the safety of the location of services and equipment in case of floods
Financing and Resource Allocation	06 resources [11, 35, 36, 39–41]	1. Availability of mechanisms for funding climate-resilient HCF projects for newly planned improvements 2. Budget allocated by the Ministry of Health for climate change adaptation 3. Adaptation costs and potential damages from climate change are included in investment plans
Intersectoral Collaboration and Partnerships	04 resources [11, 35, 36, 40]	1. Effective cross-sector collaboration in implementing climate change and health initiatives 2. Established multi-stakeholder and inter-programmatic task force on health and climate by the Ministry of Health 3. Established partnerships between HCF, community, and local health authorities to reduce climate vulnerability
Access and Continuity of Health Services	06 resources [11, 35, 36, 39–41]	1. HCF with defined essential health services packages that prioritize services during a crisis 2. HCF with implemented health service continuity plans 3. Strengthening supply chain resilience and stockpiling essential supplies
Water, Sanitation, and Hygiene (WASH)	05 resources [11, 35, 36, 38, 42]	1. Water quality is monitored regularly, including in emergencies 2. WASH climate risks management plan implemented 3. Training program on WASH practices
Community Engagement and Participation	03 resources [11, 35, 36]	1. Community participation in climate resilience activities 2. Established mechanism for community feedback 3. Community education initiatives for reducing climate and health risks launched
Energy Efficiency and Renewable Energy	5 resources [11, 35, 36, 38, 39]	1. Assessed energy needs, availability, and alternative cost-effective sources of renewable energy 2. Prioritised energy-saving measures that are least costly to introduce and bring the biggest savings 3. HCF’s fossil fuel consumption is reduced by using alternative renewable energy sources
Healthcare Waste Management and Recycling	05 resources [11, 35, 36, 38, 42]	1. Implementation of waste management practices 2. Training programs on waste management 3. Established building regulations and waste management infrastructure
Climate Research	03 resources [11, 35, 36]	1. Research findings are effectively communicated and translated into practice through knowledge management platforms 2. National research priorities align with guidance from global and regional research agendas 3. Integration of research findings and iterative learning into disaster risk reduction strategies in response to climate extremes
Reducing GHG Emissions	01 resources [11]	1. Mechanism to estimate GHG emissions in the health system established 2. Assessment of health sector GHG emissions conducted 3. Agreements with health system suppliers to reduce GHG emissions in the supply chain have been established

### Barriers to building climate resilience in HCFs inLMICs

Health systems and facilities in LMICs face numerous barriers to building climate resilience. The included resources identified and analyzed these barriers across multiple domains, revealing variations in how frequently they were discussed in the literature. These can be broadly grouped as frequently reported, moderately reported, and context-specific or emerging barriers.

**Frequently reported barriers** discussed across the reviewed literature were in the areas of policy, planning, and leadership; disaster preparedness and surge capacity; WASH; energy systems; and climate research. In policy and planning, short-term emergency responses dominate, with limited attention to long-term scenario planning and the development of comprehensive climate-health policies [46, 47]. The absence of context-specific adaptation strategies [48] and a limited understanding of resilience further complicate the efforts [41, 49, 50]. Disaster preparedness is hindered by insufficient surge capacity [41, 51], inadequate preparedness plans [35, 52–54], and a lack of comprehensive models for evaluating resilience [55]. Many facilities also face an inadequate water supply and poor sanitation practices, increasing their vulnerability to climate impacts [53, 56, 57]. Energy-related barriers include low adoption of renewable technologies and dependence on unreliable electricity sources, leading to inefficiency and operational disruptions [4, 16, 38, 47, 58]. Additionally, limited climate research and the absence of locally relevant evidence hinder informed decision-making and the development of tailored adaptation strategies.

**Moderately reported barriers** were observed in health workforce capacity, financing, and resource allocation, service delivery, infrastructure design, and healthcare waste management. In the health workforce domain, in HCFs, there is a lack of trained professionals with expertise in climate resilience [4, 16, 52, 56, 59], while infrastructure design fails to incorporate climate resilience adequately, leaving facilities vulnerable to climate-related shocks [4, 24, 43, 47, 53]. Financing gaps, including insufficient funding [16, 37, 52], and a lack of financial planning further exacerbate the challenges [60]. In terms of access to services, HCFs struggle with ensuring consistent availability of medicines and maintaining essential services during climate hazards [4, 47]. Furthermore, inadequate healthcare waste management practices, including inappropriate incineration, contribute to greenhouse gas emissions and environmental pollution [4, 16, 57].

**Context-specific or emerging barriers** appeared less frequently in the reviewed resources, but highlight systematic and social gaps. Weak intersectoral collaboration and partnerships [53], characterized by poor coordination between sectors [46, 50, 52], and a limited multi-sectoral approach to resource planning [61], constrain integrated responses. Similarly, community engagement is limited, with low awareness and trust in government programs [47, 62]. Data management is hampered by poor data collection systems, inconsistent data, and limited coordination with meteorological services, leading to gaps in climate resilience assessments [61].

## Discussion

This scoping review identified 33 resources to build climate resilience in HCFs in LMICs. These resources include academic articles, guidelines, frameworks, toolkits, reports, manuals, and books. The resources are diverse and include various plans, interventions, strategies, and indicators for climate resilience.

Typology of Resources: Publications on this topic have increased since 2020 [63], indicating a growing interest in building climate resilience in healthcare systems and facilities. Most resources identified are designed for global use rather than specifically for LMICs or HICs. While many WHO-developed resources are meant to be flexible and adaptable to local requirements, some users may find that they do not fully meet their specific needs or context. The WHO guidance needs to be more broadly implemented and tested in LMICs to evaluate its applicability and feasibility in building climate resilience in HCFs. These resources can provide a starting point for building climate resilience in HCFs [14]. However, effective use requires training for healthcare staff and education [59]. Healthcare managers or planners need to integrate climate resilience-building approaches with carbon literacy and the ability to understand climate change projections; however, this may require a large learning curve [64]. There are very few resources specifically developed by LMICs with applications in LMICs [38, 57]. Furthermore, we found resources that were mainly focused on the system-level [65], with fewer resources designed for HCFs.

Only 36% of the resources identified in this review were classified as originating in LMICs, based on the institutional affiliation of the authors. We acknowledge that author affiliation is an imperfect proxy for the development context of a resource and does not necessarily indicate whether meaningful consultation or co-production with LMIC stakeholders occurred during the development process. However, this pattern still highlights a structural imbalance in visible leadership and authorship. While global and high-income country frameworks provide broad strategic guidance, they may lack the contextual specificity required to address locally embedded vulnerabilities, governance constraints, and resource limitations in LMIC health systems. Conversely, although fewer in number, LMIC-generated resources tended to reflect operational realities and locally adapted solutions. This suggests that increasing locally led development and co-production processes is critical to ensuring that global guidance is both relevant and implementable across diverse LMIC contexts.

The identified resources also varied considerably in their methodological rigor, evidence base, and readiness for use in LMICs. While conceptual frameworks, such as the WHO Operational Framework (2023), provide valuable structure, they often remain high-level and require contextual adaptation to meet HCF-level realities. In contrast, toolkits and checklists developed or applied in LMICs, such as those from Fiji [38] and the Philippines [57], demonstrate greater operational relevance but limited generalizability beyond their local settings. Overall, the evidence base remains uneven, with few resources reporting measurable outcomes or validation. This imbalance highlights the need for implementation research to assess usability, effectiveness, and scalability within diverse LMICs’ health systems contexts. There is, therefore, a need for LMICs to develop and tailor approaches for building climate resilience based on local applications [66, 67], and develop new approaches if needed [68].

Interventions: Our review identified several domains of interventions within existing resources to build climate resilience in HCFs, aligning with the ten components of the WHO operational framework for building climate-resilient and low-carbon health systems [11]. These domains represent the essential categories of adaptation areas that enable HCFs to anticipate, prepare for, respond to, and recover from climate-related events, ensuring continuity of health service delivery by trained health workforce in climate-resilient HCFs [4]. Domains, such as climate research [14, 69], and reducing GHG emissions, appear in only a few resources, despite their long-term significance for sustainable resilience. These domains remain underemphasized in LMICs and require greater attention in future planning, given their important links with low-carbon actions [16]. The 2015 Paris Agreement (Article 7) established a global adaptation goal to enhance adaptive capacity, strengthen resilience, and reduce vulnerability to climate change, aligning with the Sustainable Development Goals. To clarify and track progress, a work program launched at COP26 produced a framework at COP28 [70] with defined climate adaptation and resilience targets, guiding countries in strengthening their climate defences. A two-year program was also initiated to develop indicators for assessing progress, supported by technical experts [71].

To conceptualize this, the Glasgow-Sharm el-Sheikh work program was launched at COP26 (2021), under the Subsidiary Bodies for Scientific and Technological Advice and Implementation. In 2023, efforts focused on finalizing this framework, leading to CMA 5’s adaptation of the UAE framework for global climate resilience. CMA 5 also initiated a two-year UAE -Belém work program to develop progress indicators, with expert support facilitated by SB 60 [71].

Indicators: Indicators identified in the reviewed resources help confirm how climate resilience interventions improve health outcomes, supported by evidence [72]. They are essential for policymakers and practitioners to design, evaluate, and refine planning and implementation efforts [73]. Without clear indicators, it is difficult to develop and test interventions for building climate resilience in health systems [72]. Despite numerous frameworks, few evaluations of implemented interventions exist, indicating a gap between theory and practice [8]. Health adaptation is becoming progressively more difficult for authorities because of increased events, complexity of impacts, increased demand for action, and competition for resources [11, 74]. Monitoring climate risks to individuals and health systems is therefore important for adaptive management, reducing uncertainties, and allowing health systems to adjust as needed [75].

Although resources on climate resilience are increasing, no standardized set of indicators exists to guide multidimensional policy and practice [76]. Robust indicators should monitor vulnerability, risks, exposures [76, 77], and impacts on HCFs [14], health systems, and populations [36, 78]. In LMICs, indicator development needs to be context-specific, reflecting each region’s unique challenges and capacities. There is no universal set; indicators are required to be tailored [79] to national and subnational needs [37], integrating with existing systems like national health surveillance [36].

The indicators identified by this review are insufficient to accurately monitor health system or facility resilience amid evolving climate risks [75] and do not reflect actual progress toward sustainable healthcare [80]. Most are static and cannot measure HCF resilience in a dynamic way, which depends on process-based, adaptive responses to disruptions [81]. While the WHO has developed indicators and checklists in its recent frameworks, these may not be suitable for all contexts, highlighting the need for adaptable, robust indicators [80]. This lack of consistent indicator standards makes it difficult to compare climate resilience across different situations or contexts.

The WHO Framework for Measuring the Climate Resilience of Health Systems [36] offers a starting point for indicator development, focusing on upstream determinants of exposure, vulnerability, system functions, and outcomes. New indicators need to rely on local data to evaluate the ability of health systems and HCFs to anticipate and manage climate risks. This may involve vulnerability assessments using the WHO’s 2021 checklist [45] and updating adaptation plans based on recent and context-specific climate data. Alongside vulnerability, capacity, and adaptation assessments, climate stress tests are needed to evaluate and strengthen the resilience of HCFs in LMICs [51]. In resource-limited settings, indicators can track changes over time [75, 82], adjusting strategies, and addressing gaps to enhance climate resilience [83] of HCFs against climate-related shocks and stresses.

Barriers: The resources highlight several barriers to preparing HCFs in LMICs for climate change. These include a lack of context-specific adaptation strategies, insufficient preparedness plans, and an emphasis on short-term emergency responses in policy, planning, and leadership. This focus on immediate actions often hinders long-term scenario planning and the development of comprehensive climate-health policies, presenting significant challenges to building effective climate resilience in HCFs [35, 46, 47]. Resources exist to guide policy and planning for climate resilience-building activities in HCFs [4]. However, many are based on top-down approaches where legal obligations at sub-national levels are overlooked [84]. Gaps in implementing existing policies at the grassroots level hinder adaptation efforts, while disconnects between national, regional, and local levels [85] and weak feedback mechanisms [52] further exacerbate these challenges. Implementation needs to be adaptive and continually adjusted to local conditions [86]. Coordination and policy coherence are also hampered by unclear responsibilities, limited awareness, and low political commitment [37, 84, 87].

The identified resources provide limited guidance on coordination, collaboration, and feedback mechanisms among stakeholders. This gap complicates communication, as participants often have diverse backgrounds [88], mandates, and terminologies [52]. Consequently, complex coordination processes and limited capacity create barriers even in countries with established governance frameworks [84]. Strengthening local, regional, and sub-national obligation [89], could accelerate implementation of adaptation policies [84]. However, operationalizing these policies requires substantial funding, which is often insufficient in LMICs. The 2020 Lancet Countdown on Health and Climate Change Report estimated that while 50% of countries had health and climate strategies, only 9% had adequate financing for implementation [90].

In LMICs, applying available guidelines may require significant resources [9], expertise [49, 68], and time. The complexity, data needs, and technical requirements of many frameworks make their use challenging in LMICs. Most resources in the scoping review emphasized physical infrastructure resilience [4, 65, 91], often neglecting social and community resilience. Adapting health systems to climate change remains difficult in resource-limited contexts, but fostering stakeholder engagement and bidirectional communication can strengthen the shift from reactive to anticipatory adaptation [48].

Implications and Way Forward: This review underscores that while global frameworks provide a robust foundation, their practical application in LMICs requires contextualization, simplification, and capacity building. Future work needs to prioritize co-creation of tools with local stakeholders’ involvement, alignment with national adaptation priorities, and integration of climate-resilience indicators into health information systems. Establishing regional collaborations and peer learning mechanisms could accelerate progress, allowing LMICs to adapt and refine frameworks based on shared experience.

The limitations of this review include potential publication and selection biases. The search strategy was restricted to English-language publications, potentially overlooking evidence published in local languages in LMICs. A publication bias may also exist due to the barriers that LMIC authors face in academic publishing. Moreover, although the review aims to identify resources for building climate-resilient HCFs in LMICs, most available resources were developed for global application, offering limited guidance for local adaptation and implementation. This lack of contextualization is a methodological constraint, underscoring the need for further research on local applicability, feasibility, and equity. Furthermore, although some quantitative counts were provided, the conceptual and heterogeneous nature of the included material constrained detailed quantitative synthesis. Therefore, this review prioritizes thematic and descriptive mapping, which may reduce comparability but provides a meaningful synthesis of diverse evidence. Additionally, since this review followed a scoping review methodology, the focus was on synthesizing available resources rather than critically evaluating or scoring their quality.

## Conclusion

This scoping review identified 33 diverse resources aimed at enhancing the climate resilience of HCFs in LMICs. While these resources are generally global in scope, their effective applications rely on adapting to the specific contexts of LMICs, including the unique needs of local populations, settings, and broader health systems. Despite the availability of these resources, LMICs face significant challenges in implementing and evaluating climate resilience programs due to high vulnerability and limited adaptive capacity. This review addresses these gaps by offering a comprehensive typology of resources and a structured framework of 14 domains with associated indicators to guide and assess climate resilience efforts in HCFs, supporting more targeted and effective climate adaptation efforts.

The synthesis of barriers and recommendations in this review emphasizes the importance of context-specific, collaborative approaches to overcome challenges in building climate resilience. By presenting action-oriented resources and measurable indicators, the review strengthens the capacity of stakeholders in LMICs to develop adaptive, resilient health systems, contributing to global efforts to protect healthcare services amid climate change impacts.

Future research should focus on implementation science to explore best practices for adapting these resources to various local contexts in LMICs. Additionally, there is a need for evaluation research to assess the effectiveness of climate resilience strategies over time and under different climate stressors. Developing and testing robust feedback mechanisms across national, regional, and local levels can enhance coordination among stakeholders and ensure that climate resilience policies are responsive to the evolving needs of HCFs in LMICs. Ultimately, advancing these research areas will contribute to a more comprehensive understanding of what drives successful, sustainable climate resilience in healthcare systems globally.

## Supplementary Information

Below is the link to the electronic supplementary material.

Supplementary Material 1

## Data Availability

No datasets were generated or analysed during the current study.

## Abbreviations

HCF: Healthcare facility
HCFs: Healthcare facilities
LMICs: Low and middle-income countries
MICs: Middle-income countries
HICs: High-income countries
LICs: Low-income countries
LDCs: Least developed countries
WASH: Water, sanitation, and hygiene
PRISMA: Preferred reporting items for systematic review and meta-analysis
PICO: Population, interventions, comparison, and outcome
PIO: Population, intervention, and outcome
GHG: Greenhouse gas
COP: Conference of the parties
WHO: World Health Organization
PAHO: Pan American Health Organization
GCF: Green climate fund
GAMI: Global adaptation mapping initiative
HCWH: Healthcare without harm
GEF: Global environmental facility
CMA: Conference of the parties serving as the meeting of the parties to the paris agreement
SB 60: 60th session of the subsidiary bodies
