# Supplementary material for: Towards resilience: investigating resources for enhancing climate resilience in health care facilities in low and middle-income countries. a scoping review
Source: BMC Health Serv Res. 2026 Feb 6;26:311. doi: 10.1186/s12913-026-14089-x (PMC12937575; doi:10.1186/s12913-026-14089-x)
Supplement: Supplementary file 1 — Supplementary Material 1 [file 12913_2026_14089_MOESM1_ESM.docx]

**Supplementary Table: Typology of included articles**

| **S.**  **No:** | **Resources Citation (author, date)** | **Resources description** | **Audience** | **Length** | **Practical features** | **Structure:** | **Output** |
| --- | --- | --- | --- | --- | --- | --- | --- |
| 1 | Operational framework for building climate-resilient health systems  (World Health Organisation 2015)(36). | Framework for enhancing health systems’ resilience to climate variability. | Health professionals, health managers, policymakers, and decision-makers. | 47 pages | Framework for Strengthening Health Resilience to Climate Change. | Building block-based. | Ten components and six building blocks for enhancing climate resilience of health systems. |
| 2 | Climate Sensitive Adaptation in Health  Imperatives for India in a Developing Economy Context. (Dasgupta 2016)(62). | This book provides an in-depth analysis of climate-sensitive health risks in India and explores adaptation strategies within the context of developing economies. | Public health planners, researchers, policymakers, and academicians. | 194 pages | Interdisciplinary approach and evidence-based narrative. | Adaptation measures. | Impacts of climate change on health, adaptation strategies, policy analysis, and case studies. |
| 3 | Enhancing the sustainability and climate resiliency of health care facilities: a comparison of initiatives and toolkits (Balbus et al. 2016)(60). | Enhancing the sustainability and climate resiliency of HCFs by comparing three initiatives and toolkits designed to prepare for and mitigate the impacts of climate change. | Healthcare officials, policymakers, and health facility designers. | 07 pages | Resiliency initiatives, toolkit comparisons. | Hazards-based. | Comparison of initiatives and toolkits. |
| 4 | Smart Hospitals Toolkit (Pan American Health Organization 2017)(44). | The toolkit is designed to build HCFs that are safe and environmentally friendly, focusing on resource conservation, cost reduction, operational efficiency, and reduction in carbon emissions. | Hospital administrators, health facility designers, & professionals. | 152 pages | Strategies for smart HCFs. | Hazards and building block-based. | The Hospital Safety Index, Baseline Assessment tool, and Green Guide. |
| 5 | Understanding Weather and Hospital Admissions Patterns to Inform Climate Change Adaptation Strategies in the Health Sector in Uganda (Bishop-Williams et al. 2018)(49). | The article provides insights into weather patterns and hospital admissions to inform adaptation strategies. | Researchers, policymakers, & health service providers. | 14 pages | Hospital Planning and Preparedness based on hospital admission patterns with seasonal variability. | Building block-based. | It utilizes existing data sets for hospital and meteorological data to inform climate change adaptation strategies in the healthcare sector. |
| 6 | Stress Testing the Capacity of Health Systems to Manage Climate Change- Related Shocks and Stresses (Ebi et al. 2018)(52). | The article discusses the development of climate and health stress tests to enhance the resilience of the health system against climate change-related shocks and stresses. | Healthcare professionals, policymakers, researchers,  Health administrator. | 16 pages | stress testing to evaluate the resilience of health systems. | Building block-based. | Preparation of stress testing  Conduction of stress test.  Communication of results to key stakeholders. |
| 7 | Caribbean action plan on health and climate change (PAHO 2019)(40). | The documents outline methods for measuring and reporting on various health and climate change indicators. | Caribbean health leaders and policymakers. | 23 pages | Indicators for climate resilience. | Strategic lines of action. | Indicators descriptions. |
| 8 | Developing a practical toolkit for evaluating hospital preparedness for surge capacity in disasters (Shabanikiya et al. 2019)(56). | Developing a toolkit for evaluating hospital preparedness for surge capacity in disasters. | Experts in hospital surge capacity, healthcare managers, and hospital administrators. | 06 pages | Scoring system and 05 level of preparedness. | Building block-based. | The toolkit consists of 64 components in five categories and 13 subcategories. |
| 9 | Are we already for it? Health systems preparedness and capacity towards climate change-induced health risks: perspectives of health professionals in Ghana (Hussey et al. 2019)(57). | The preparedness and capacity of health systems and professionals in Ghana to manage climate-induced health risks. | Health professionals, policymakers, researchers, and public health sectors. | 14 pages | Discussing the preparedness and capacity of health systems. | Building block-based. | Perceptions of health professionals.  Knowledge gaps,  Recommendation of policy and action. |
| 10 | WHO Guidance for Climate-resilient and Environmentally Sustainable Healthcare Facilities (World Health Organization 2020)(4). | The guidance provides a framework for enhancing the climate resilience and environmental sustainability of HCFs to ensure effective healthcare services. | Health professionals, policymakers, and HCF managers. | 79 pages | Recommendation for climate and sustainable HCFs. | Building block-based. | Outputs include checklists, lists of actions, and case studies as examples of good practices. |
| 11 | Guidelines for Climate Resilient and Environmentally Sustainable HealthCare Facilities in Fiji (Ministry of Health and Medical Services 2020)(39). | Strategies for building climate-resilient and environmentally sustainable HCF in Fiji, focusing on practical actions across various domains to ensure the health sector contributes positively to climate resilience. | Health professionals, policymakers, and facility managers. | 58 pages | Measures for climate-resilient and environmentally sustainable HCFs. | Building block and hazard-based. | Checklist, Assessment procedure, standard operating procedures, and format for assessment report. |
| 12 | Defining adaptive capacity in healthcare: A new framework for researching resilient performance (Anderson et al. 2020)(50). | The article presents an integrated resilience attributes framework for researching resilient performance in healthcare, focusing on anticipating, monitoring, responding, and learning. | Researchers, health care professionals, policymakers, and regulators. | 09 pages | Framework for studying adaptive capacity in healthcare. | it integrates three theoretical perspectives. | Integrated Resilience Attributes Framework. |
| 13 | A checklist to improve health system resilience to infectious disease outbreaks and natural hazards. (Meyer et al. 2020)(45). | The article presents a health system resilience checklist designed to measure and improve the readiness of health systems to effectively respond to infectious disease outbreaks and natural hazards while maintaining essential healthcare services. | Health system managers, health facility managers, Health system stakeholders, and researchers. | 07 pages | Health system resilience checklist, which includes ten thematic categories. | Building block-based. | Resilience checklist & thematic categories. |
| 14 | Understanding the resilience of health systems (Blanchet et al. 2020)(51). | This chapter discusses how health systems can adapt and transform in response to challenges. | Health system researchers, health systems managers, health practitioners, and policymakers. | 22 pages | Conceptual framework based on systems. | Conceptual framework. | A proposed framework for the analysis of resilience of health systems with four main dimensions of resilience. |
| 15 | Impact of extreme weather conditions on healthcare provision in urban Ghana (Codjoe et al. 2020)(54). | This article discusses the vulnerabilities of Ghana’s urban healthcare system to extreme weather events and explores resilience strategies by service providers and communities. | Healthcare providers, community members, researchers, academicians, and policymakers. | 12 pages | Strategies for resilience building. | Hazards-based. | Investigating health systems vulnerabilities. |
| 16 | Towards Climate Resilient and Environmentally Sustainable Health Care Facilities (Corvalan et al. 2020)(16). | A framework for enhancing the climate resilience and environmental sustainability of HCFs. | Healthcare professionals, policymakers, and health sector officials. | 18 pages | Practical framework. | Hazards-based. | Framework with key areas of action. |
| 17 | Resilience Testing of Health Systems: How Can It Be Done? (Rogers et al. 2021)(41). | It presents a five-phased approach and methodology to assess and enhance health system resilience through participatory processes and scenario analysis. | Hospital managers, senior clinicians, policymakers, health authorities, and researchers. | 17 pages | Resilience test toolkit, 05 phased approach to implementing resilience testing. | Multi-dimensional health and social care system (MHSCS). | The resilience testing concept includes a toolkit. |
| 18 | Climate Change and Health Vulnerability and Adaptation Assessment (Health Canada and World Health Organization 2021)(38). | The document is for assessing the vulnerability of health systems to climate change and developing adaptation strategies to enhance their resilience. | Health professionals. Policymakers, researchers, and cross-sectoral entities. | 70 pages | Vulnerability and adaptation assessment. | Building block-based. | Vulnerability and adaptation assessment. |
| 19 | Quality Criteria for Health National Adaptation Plans (World Health Organization 2021) [50]. | Quality criteria and guidance for developing national health adaptation plans to address the health impacts of climate change. | Ministries of health, health sector stakeholders, and cross-sectoral partners. | 30 pages | Guiding principles, case studies, and practical applications. | Building block-based. | Six Quality Criteria. |
| 20 | Checklists to assess vulnerabilities in healthcare facilities in the context of climate change (World Health Organization 2021)(46). | This resource provides checklists to assess vulnerabilities in HCFs in the context of climate change focusing on risks, impacts, and resilience. | HCFs managers, health workers, partners in other sectors, and local and national government agencies. | 97 Pages | Comprehensive checklists and targeted assessments, guidance for actions, and adaptability. | Hazards-based. | Checklists for climate hazards: floods, storms, sea level rise, droughts, heat waves, wildfires, and cold waves. |
| 21 | Green and Safe Health Facilities Manual (Department of Health Manila 2021)(58). | It is developed to address and adapt to the impacts of climate change and to minimize carbon footprints while continuing to provide quality health services. | Medical directors, technical professionals, developers, contractors, healthcare and hospital administrators. | 141 pages | climate-smart adaptation, mitigation measures, and preparedness. | Building block-based. | Green and safe health facility interventions. |
| 22 | Advanced Operationalization Framework for Climate-Resilient Urban Public Health Care Services: Composite Indicators-based Scenario Assessment of Khon Kaen City, Thailand (Puntub and Grieving 2022)(47). | An operationalization framework for enhancing the climate resilience of urban public healthcare through the use of composite indicators and scenario assessment. | Academic researchers, public health officials, urban planners, and policymakers. | 20 pages | Framework, composite indicators, and scenario planning. | Hazards-based structure. | Composite indicators and scenarios planning. |
| 23 | 360$^{\circ}$ Resilience  A Guide to Prepare the Caribbean for a New Generation of Shocks (Julie Rozenberg et al. 2022)(53). | The guide provides strategies and recommendations for Caribbean nations to enhance their resilience against a variety of economic and natural shocks, with a focus on improving government efficiency, empowering households and the private sector, and reducing physical risks. | Policymakers, resilience experts, and decision-makers. | 392 pages | Government efficiency, empowerment, physical risk reduction, and digitalization. | Building block-based. | A diagnosis, a progress assessment, and an analysis of the resilience levels using a traffic light system approach. And a way forward. |
| 24 | Health systems resilience toolkit (World Health Organization 2022)(63). | The toolkit is designed to support the strengthening and resilience of health systems across various contexts, focusing on policy-making, operationalization, and monitoring and evaluation. | Policymakers, health authorities, health services providers, managers, and technical partners. | 70 pages | The toolkit provides four interconnected modules and technical resources. | Four modules | Four modules for building health system resilience. |
| 25 | Measuring the climate resilience of health systems (World Health Organization 2022)(37). | It provides a framework and suggested approach for measuring the climate resilience of health systems. | Health authorities, local to the national level, health decision-makers, and health professionals. | 35 pages | Actions and indicators. | Building block-based. | Indicators for measuring the climate resilience of the health system. |
| 26 | WASH FIT  A practical guide for improving quality of care through water, sanitation, and hygiene in healthcare facilities (WHO and UNICEF 2022)(43). | It provides a comprehensive framework for WASH for improving water, sanitation, and hygiene in HCFs to enhance infection control, patient safety, and quality of health care services. Prioritize specific WASH actions that are climate-resilient, equitable, and inclusive. | HCFs managers, policymakers, government officials, and quality improvement teams. | 119 pages | WASH FIT is a risk-based management tool for HCFs. | Risk-based management tool. | Guide for improving the quality of WASH. |
| 27 | Operational framework for building climate resilient and low carbon health systems (World Health Organization 2023)(11). | An update on the 2015 operational framework, including low-carbon solutions. It is a framework to increase the climate resilience of health systems and to protect and improve the health of communities while optimizing the use of resources and reducing GHG emissions. | Health sector managers, public health professionals, decision-makers, policymakers, and organizations involved in climate resilience. | 84 pages | Operational Framework for building climate-resilient and low-carbon health systems. | Building block-based. | Ten components are linked to six building blocks. |
| 28 | STAR-H Strategic Toolkit for Assessing Risks in Health Facilities (Pan American Health Organization 2023)(25). | The STAR-H methodology is a toolkit designed to help HCF assess and manage risks from various hazards, enhancing their preparedness and response capabilities. | HCF personnel with duties related to management, planning, response, coordination, and incident command. | 29 pages | Multi-Hazards approach. | Hazards-based approach. | STAR-H for risk assessment of a health facility. |
| 29 | Operational Guidelines  Green, Safe, and Climate-Resilient Health Facilities (Department of Health-Health Facility Development Bureau, Philippines 2023)(15). | Provides operational guidelines for green, safe, and climate-resilient HCFs. | HCF administrators, health care providers, and technical experts | 11 pages | Operational guideline focusing on the climate resilience of HCFS | Building block-based. | Green viability assessment tools, performance standards, rating systems, and assessment process. |
| 30 | Exploring hospitals’ functional preparedness effective factors in response to disasters: a qualitative study in a lower-middle-income country. (Samei et al. 2024)(55). | A qualitative study that explores the factors influencing hospital preparedness for disasters in LMICs. | Hospital managers, decision-makers, and researchers. | 14 pages | Hospital functional preparedness in LMICs. | Both hazard-based and building block-based. | Qualitative study for hospital preparedness in LMICs. |
| 31 | Iran’s climate resilient health system: Challenges and solutions (Mosadeghrad et al. 2024)(48). | This resource discusses the challenges and solutions for strengthening the climate resilience of Iran’s health system. | Policymakers, health managers, healthcare providers, health experts, and researchers. | 34 pages | The challenges and solutions to build a climate-resilient health system. | Both are hazards and building block-based. | Qualitative study. |
| 32 | Strengthening Health Systems  A Practical Handbook for Resilience Testing (OECD 2024)(42). | It outlines a collaborative testing methodology for assessing the vulnerabilities of health systems to specific shocks. | Anyone planning to undertake a health system resilience test. | 220 pages | Resilience testing in a wide range of shock scenarios. | Building block-based. | Resilience testing methodology. |
| 33 | Global Framework for Action 2024-2030  Universal water, sanitation, hygiene, waste, and electricity services in all healthcare facilities to achieve quality healthcare services (World Health Organization 2024)(59). | This framework deals with the WASH, waste, and electricity elements of the WHO comprehensive approach to building safe, climate-resilient, and environmentally sustainable HCFs. | Health leaders, program managers, policymakers, technical experts, development partners, financial institutions, and civil society. | 24 pages | Framework | Building block-based. | Framework for implementing the WASH, Waste, and electricity services in HCFs. |
